# Inferring novel disease indications for known drugs by semantically linking drug action and disease mechanism relationships

**DOI:** 10.1186/1471-2105-10-S5-S4

**Published:** 2009-05-06

**Authors:** Xiaoyan A Qu, Ranga C Gudivada, Anil G Jegga, Eric K Neumann, Bruce J Aronow

**Affiliations:** 1 Department of Biomedical Engineering, University of Cincinnati, Cincinnati, OH, USA; 2 Department of Pediatrics, University of Cincinnati, Cincinnati, OH, USA; 3 Division of Biomedical Informatics, Cincinnati Children's Hospital Medical Center, Cincinnati, OH, USA; 4 Procter & Gamble Pharmaceutical, Cincinnati, OH, USA; 5 Clinical Semantics Group, Lexington, MA, USA

## Abstract

**Background:**

Discovering that drug entities already approved for one disease are effective treatments for other distinct diseases can be highly beneficial and cost effective. To do this predictively, our conjecture is that a semantic infrastructure linking mechanistic relationships between pharmacologic entities and multidimensional knowledge of biological systems and disease processes will be highly enabling.

**Results:**

To develop a knowledge framework capable of modeling and interconnecting drug actions and disease mechanisms across diverse biological systems contexts, we designed a *Disease-Drug Correlation Ontology (DDCO)*, formalized in OWL, that integrates multiple ontologies, controlled vocabularies, and data schemas and interlinks these with diverse datasets extracted from pharmacological and biological domains. Using the complex disease Systemic Lupus Erythematosus (SLE) as an example, a high-dimensional pharmacome-diseasome graph network was generated as RDF XML, and subjected to graph-theoretic proximity and connectivity analytic approaches to rank drugs versus the compendium of SLE-associated genes, pathways, and clinical features. Tamoxifen, a current candidate therapeutic for SLE, was the highest ranked drug.

**Conclusion:**

This early stage demonstration highlights critical directions to follow that will enable translational pharmacotherapeutic research. The uniform application of Semantic Web methodology to problems in data integration, knowledge representation, and analysis provides an efficient and potentially powerful means to allow mining of drug action and disease mechanism relationships. Further improvements in semantic representation of mechanistic relationships will provide a fertile basis for accelerated drug repositioning, reasoning, and discovery across the spectrum of human disease.

## Background

Drug repositioning – the use of established drugs for new indications – represents a promising avenue for the development of therapeutics based on its relatively low cost and ready availability of extensive data and knowledge from prior research and development efforts [1]. Despite impressive successes shown by repositioned drugs, most of these are the result of "serendipity", i.e. based on unexpected findings made during or after late phases of clinical study. Improved ability to identify likely of these candidate new disease indications is attractive for the potential to expedite drug development process and to minimize costly clinical trials. One of the reasons that linking drug candidates and potential new applications is difficult is that mechanisms of drug action are at best only partly understood, are subject to context and individual specific variation, and importantly, tend to be poorly represented with current knowledge modeling and data representation methodologies. In biomedical literature, underlying mechanisms associated with drug action are usually buried within drug- and disease-associated narratives. Thus, the establishment of an informatics model using *in silico *approaches that could improve data capture, integration, and interpretation pertinent for the prediction of potential new therapeutic indications for drugs based on integrated biomedical knowledge around drug and disease mechanisms is highly desirable. The requirements for such an approach are to generate a comprehensive knowledge base that must both broadly and deeply represent factual knowledge and correlated phenomena from across pharmacological and biological domains. Doing this would, ideally, allow researchers, and subsequently, care providers, to distill insightful hypotheses and derive the best decisions. Computational approaches exist to allow the integration of heterogeneous data, such as by schema merging, federating databases, or by unifying data models. Notably, while great efforts have been made in connecting the biological and chemical domains, including PharmGKB [2], KEGG [3], and DrugBank [4], these databases were not designed to enable mechanism-based representation of relationships between therapeutic drugs and phenotypic features associated with health and disease. While clearly a fuzzy area, the lack of phenotypic feature relationships impedes our ability to elucidate and leverage embedded mechanistic associations. Recently, *Lamb et al *group designed a promising "connectivity map" approach which associates small molecules, genes, and diseases through genomics profiling connections [5]. This approach represents a significant advancement in linking drugs to underlying diseases. However, since these functional connections are purely based on one-dimensional data (i.e. gene expression profiling as a sole surrogate for phenotype, with limited diversity of tested cell lines and lack biological contexts), the approach is likely to require much greater depth of knowledge dimensionality and data connectivity.

Advancements in the development of Semantic Web (SW) [6] standards and technologies, including Web Ontology Language (OWL)  and Resource Description Framework (RDF) , as well as progress in corresponding database and knowledge representation technologies provide promising platforms for comprehensively integrating, analyzing, and visualizing heterogeneous high dimensional data using semantics and complexity of knowledge interoperability. The information layer of OWL defines domain knowledge using structured vocabulary and provides a mechanism for formalizing components for an ontology, such as classes, instances, and their relationships, therefore provides a computationally processable conceptual representation of our understanding of the domain knowledge. Based on the semantic definition in OWL, information resources can be denoted in RDF, a language represented in a simple statement form of triples *(<subject verb object>)*. A set of RDF statements can be represented in directed acyclic graph-like data network. We argue that by associating comprehensive biomedical information and prior knowledge around pharmacological entities (i.e. biological, chemical, and clinical processes) systematically and semantically using Semantic Web principles and technologies can facilitate knowledge discovery such as novel indications for known or novel drugs. To achieve this, a knowledge framework with adequate formalism that encapsulates broad and interdisciplinary range of concepts across pharmacological, biological, and clinical domains is needed. Conceivably, any single domain-specific ontology will not serve this purpose. The existing multi-domain ontologies or terminologies, such as UMLS [7], though providing decent framework for federating some biomedical databases, do not have sufficient coverage for the pharmacological-centric drug development area.

In this work, we devised a knowledge framework, *Disease-Drug Correlation Ontology *(DDCO), using OWL representation formalism. The DDCO is a result of manual curation and integration of relevant components from multiple existing ontologies, vocabularies, and database schemas. We used the constructed DDCO framework to support the integrated data representation needs of a set of prior knowledge sources, including data from DrugBank, EntrezGene, GO, OMIM, KEGG, BioCarta, Reactome, UMLS, and GEO. The data was semantically integrated into an RDF network as a pharmacome-diseasome knowledge base containing instance data represented as a web-like structure. Thereafter, we demonstrate how this integrated Semantic Web infrastructure supports knowledge mining and inference by presenting an application scenario that requires finding implicit associations between a compendium of drugs and the disease Systemic Lupus Erythematosus (SLE). Our goal in constructing and analyzing such a knowledge base is to learn essential elements that would provide critical power to the entire spectrum of drug R&D applications: to support new hypothesis generation, particularly for drug repositioning; as well as for novel target identification by establishing mechanism-based connectivity of drugs, diseases, genes, pathways, and continuously emerging biological systems knowledge.

## Results

### Construction of a unified disease-drug correlation ontology (DDCO)

**Figure 1:**
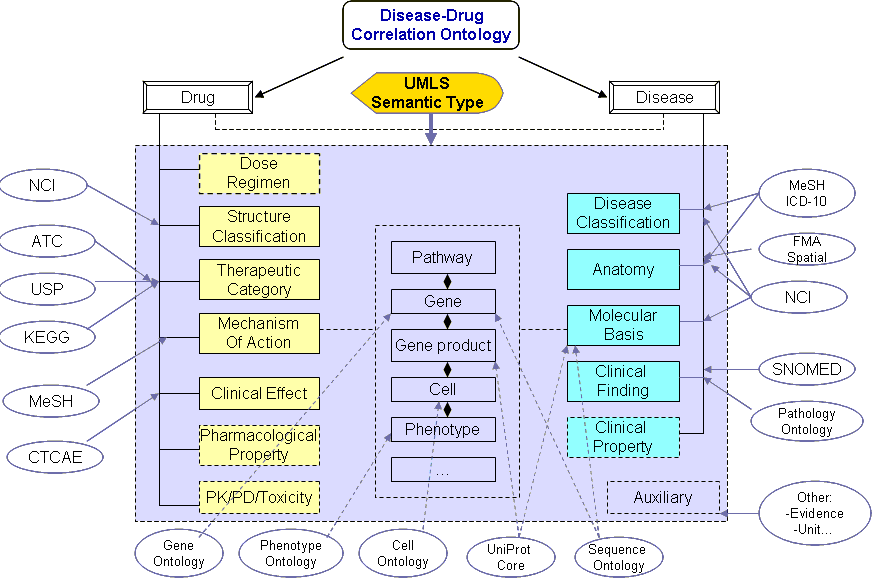
**Disease-Drug Correlation Ontology (DDCO) model**. This figure shows the schematic view of DDCO model making connections between drug and disease. The oval text denotes the major ontology or terminology sources used in constructing DDCO.

Our goal is to devise a drug- and disease-centric knowledge framework that could serve both data integration and knowledge exploration and exploitation needs. The ontology was designed with high-level of granularity and aims to reuse knowledge components whenever possible. Therefore, the first step for our ontology development effort was to construct the upper level ontology that included the basic hierarchical relations required for connecting pharmacological, clinical, and biological domains. As much as possible we used UMLS Semantic Network elements [8] to construct a scaffold for DDCO. Although UMLS Semantic Network contain a set of broad semantic types and permissible relationships among these types, with respect to disease mechanism and therapeutic agent modeling, the UMLS Semantic Network can sometimes seem to have knowledge "gaps" and difficult to follow organization [9]. For example, pertaining to therapeutic agents, the semantic type of "*Pharmacologic_Substance*" has only a single child term (e.g. "*Antibiotic*") whereas "*Molecular Mechanisms of Pharmacologic Action", "Pharmacologic Actions"*, and "*Chemical Actions and Uses*", are concept instances that are not represented as semantic types. They belong to a semantic type called "*Natural Phenomenon or Process*" which is not however semantically related to either the semantic type of "*Clinical_Drug*" or "*Pharmacologic_Substance*". Thus, at least one of the challenges in linking these terms in an ontology to enable reasoning is to provide connector relationships that facilitates domain representation, in this case principles of drug action. In this work, we have designed three key subdomains in the DDCO and created relevant knowledge components in each subdomain: 1) Pharmacological subdomain: focusing on defining concepts around drug and compound with classes such as *Mechanism_of_Action, Chemical_Substance, Manufactured_Object, Therapeutic_Category, Structure_ Classification, Pharmacologic_Property, etc*. 2) Phenomical subdomain: focusing on defining concepts and relations for disease and its associated clinical features and including classes such as *Disease_or_Syndrome, Clinical_Property, Clinical_Finding, Phenotype_Trait, Diagnosis, Etiology, etc*. 3) Biological subdomain: focusing on defining biological entities, events, and mechanisms, including key components such as *BioEntity, Molecular_Basis, BioProcess, Molecular_Interaction, and BioEv*ent, etc. Among these domains, components from biological subdomain, such as pathway, gene, molecular phenotype and function, serve as key bridge for connecting components from other two subdomains (see Figure 1).

Next, we examined existing resources and selected biomedical knowledge components required to formally represent concepts in the DDCO architecture. The knowledge resources used for DDCO construction included the following major ontologies, vocabularies, and terminologies:

• MeSH (medical subject headings) [10]: the National Library of Medicine's controlled vocabulary thesaurus, consisting of terms and headings in biomedical fields

• NCI Thesaurus [11]: an ontology-like vocabulary that has broad coverage in cancer-centric disease areas

• ATC System [12]: The Anatomical Therapeutic Chemical Classification is a WHO (world health organization) recommended classification system for internationally applicable methods for drug utilization research

• KEGG Drug Category: A chemical structure based information resource for approved therapeutics with classifications for drugs

• Common Terminology Criteria for Adverse Event (CTCAE) [13]: A descriptive terminology and grade scales adopted by NCI for drug adverse event

• Gene Ontology [14]: Controlled vocabulary published by Gene Ontology (GO) Consortium to describe gene and gene product attributes

• SNOMED CT [15]: clinical health care terminology and infrastructure

Some of these ontologies or vocabularies are independent and ready for direct integration (such as Gene Ontology), yet many resources contain overlapping and intertwining classes. For example, MeSH, KEGG Drug Category, and NCI Thesaurus all contain hierarchical and categorized terminologies which may represent *Drug *class. The *"Chemicals and Drugs" *in MeSH covers a mixture of information in a loosely-structured manner for drugs ranging from chemical structure, pharmaceutical preparation, and pharmacological actions. The *"Pharmacological Substance" *in NCI Thesaurus, however, is focused on therapeutic classifications of cancer drugs. KEGG Drug Category is also focused on therapeutic classification but is better organized to cover both application- and target-driven categories which fit general class of drug concepts including non-cancer drugs. To maximize the value of these relevant components, we chose to extract the *"Pharmacological_Action" *from MeSH and mapped it as a subclass of Drug class in the DDCO. By using ontology-merging and aligning techniques (see Methods), we populated *"Therapeutic_Category" *using knowledge components from both NCI Thesaurus and KEGG Drug Category. The *target-based *and *structural-based *categorizations from KEGG and NCI Thesaurus were also extracted and created as subclasses to represent *Drug *entity from respective aspect. In addition, we also integrated the ATC classification, a broadly accepted system for annotating approved drugs with classifications at main anatomy group, pharmacological property, chemical, and substance levels. To establish the relations between different drug classifications in the DDCO and adopted ATC system, explicit OWL class definitions were created to formally record the equivalence (or difference) among these classes.

**Figure 2:**
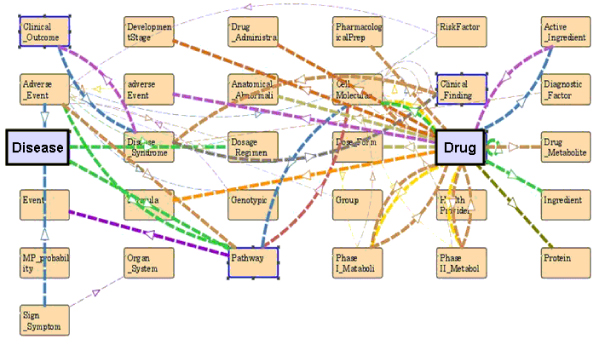
**Conceptual frames and domain/range relationships in DDCO**. This figure shows the top-level abstraction view of the major classes relevant to drug and disease classes as well as their associations defined by restrictions using property attributes.

In order to comprehensively represent key entities and relations in DDCO, we defined classes that attempted to fill in knowledge gaps (*e.g. areas lacking high quality knowledge representation or missing standards*) that could serve data integration needs. For example, we created hierarchical classes of *Pharmacokinetics *with descriptions of both ADME and toxicity features. We also generated classes to unify drug administration properties, such as preparation, drug type, and dosing and regimen (i.e. administration route, dosing interval, etc). Figure 2 presents an example of top-level view of *Drug *class as well as the key entities associated with it by defined relations. While efforts to expand and refine the conceptualization are continuing, the current DDCO contains 2046 classes (excluding GO which was imported directly), with average sibling number of 17 (maximum 35 and minimum 1) per class.

**Figure 3:**
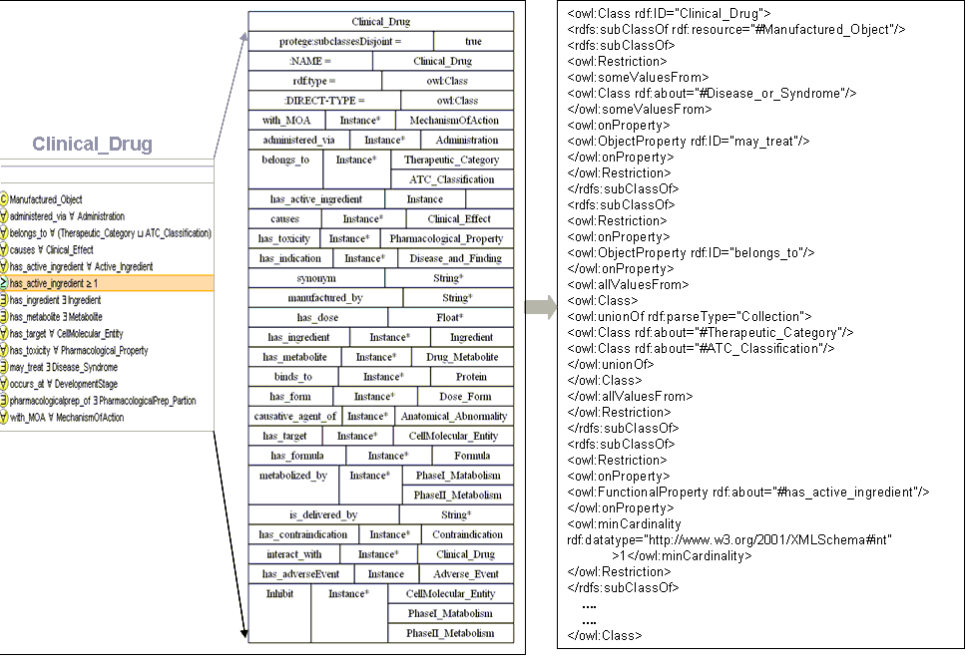
**Partial view of Clinical_Drug class modeling in DDCO**. *Left panel: *conceptual view of definition and restriction for the class of "Clinical_Drug"; *Right panel: *syntax expression of modeling in OWL

One key to realize the inference power of semantic infrastructure is to have accurate and meticulous definitions of properties as well as proper restrictions (e.g. domain and range definition). We have thoroughly examined the class relationships and defined detailed properties for the classes required for our project purpose. The DDCO contains total of 221 properties, with 99 properties domain-specified, 69 range-specified and 36 inverse-specified. Among these, 106 properties were mapped to the UMLS semantic network relations, 40 mapped to SNOMED attributes, and 75 were custom-defined properties for constraint specification. In addition, we developed 67 restrictions related to data to be integrated in the semantic knowledge base, including 7 existential, 36 universal, and 25 cardinality constraints. Figure 3 illustrates the semantic model for the *Clinical_Drug *entity (i.e. clinically approved drug) with our curation of concept restrictions including necessary and sufficient restrictions. OWL-DL was used to define each relevant class and their associations. As shown in Figure 3, the OWL syntax expresses that the class of *Clinical_Drug *is a subclass of *Manufactured_Object*. As an instance of the *Clinical*_*Drug *class, a drug may treat *Diseases or Syndromes*. It can be categorized into *Therapeutic Category *which includes custom defined classification or *ATC_Classification *system. In addition, one of the obligatory criteria to define a *Clinical_Drug *entity is that it needs to have at least one active ingredient. With the robust expression power of OWL, the complex relationships for such ontological descriptions were defined in an explicit and self-descriptive manner.

### An integrated pharmacome-diseasome RDF network

A key benefit of the Semantic Web is the ability to integrate relevant data from different origins and in incompatible formats. We have used the DDCO as the knowledge framework to integrate a diverse collection of data sources across multiple domains to create an integrated pharmacome- and diseasome- network.

**Figure 4:**
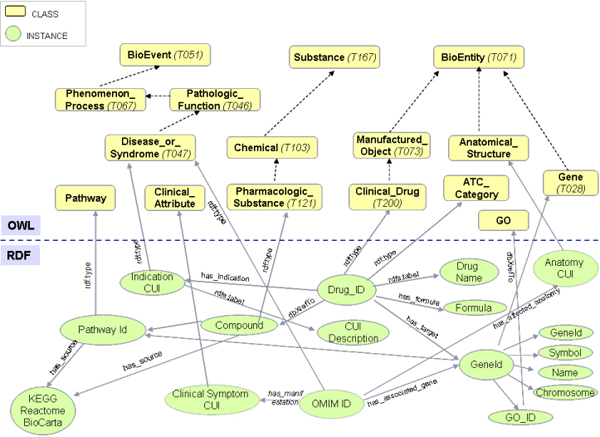
**Example of RDF data model referenced via link to DDCO in the semantic knowledge base**.

Drug-associated information was compiled from DrugBank, a database containing drug data with comprehensive target information. The dataset contains 4,763 drug entries. We parsed the information of over 1,400 FDA-approved drugs for integration in our knowledge base. Besides the information of pharmacological entity, we parsed the drug annotation and mapped the drug and drug target to associated pathways, which would allow for semantic integration with other data sources such as KEGG pathway (via mapped drug ID) and NCBI EntrezGene (via mapped gene ID). In addition, we have extracted the mapping associations between FDA-approved drugs and their indications using data from UMLS metathesaurus (see Methods). These associations, along with other entities, such as gene, pathway, drug, disease, and other molecular connectivity, were used as key linkage points to connect pharmacome and diseasome subnetworks. To generate the pharmacome network, RDF models were created in compliance with the logic and semantic definitions in the DDCO and the pharmacome data instances extracted were converted into RDF triples with designated unique name spaces. Figure 4 illustrates the relations using a schematic view of the RDF data model referenced via links to the DDCO OWL ontology. The converted RDF triples were further converted into N-Triple format using Oracle RDF loaders before loading to the Oracle 10g release 2 RDF store [16].

The diseasome network was constructed using Online Mendelian Inheritance in Man (OMIM) [17] records. Uniform Resource Identifiers (URI) derived from OMIM ID and the corresponding gene associations were used for network integration. In addition, we parsed the annotation of human genes and interactome data including an aggregation of external data sources, such as BIND [18], BioGRID [19] and HPRD [20] from NCBI EntrezGene and compiled gene-pathway annotations from KEGG, BioCarta [21], BioCyc [22] and Reactome [23] databases. The total data set contains 15,068 human genes annotated with 7,124 unique GO terms, and 14,899 gene-pathway associations. URIs derived from NIH authoritative identifiers, such as EntrezGene ID, OMIM ID, and UMLS CUI, were created for semantic integration. Similarly to the pharmacome RDF subnetwork, DDCO-compliant RDF models were created for each individual knowledge base (e.g. Gene Ontology Association, OMIM, Pathway) and RDF triples were generated using the data extracted from these knowledge sources. With the designated unique name space, the entities sharing the uniform resource identifier were collapsed and integrated when loading into the triple store.

### Topological properties of the RDF network

To further understand the topological features of our integrated RDF network, we performed degree centrality analysis on the drug-centric and gene-interaction subnetworks. Degree centrality is a network centrality measure that takes account of the degree of a node, which is the number of nodes that a given node is connected to [24]. To construct the drug-centric subnetworks, we issued RDF queries to retrieve and construct RDF graph with statements associated by "*has_target*" and "*has_indication*" properties. Similarly, we constructed the gene-interaction RDF graph for all human genes associated with their interacting genes in the integrated RDF store. The resulting interactome network consists of over 44,000 associations for 21,143 human genes. By refining pattern matching criteria in the RDF query, we further extracted and constructed two gene-interaction subnetworks for *disease genes *(consisting of 1,190 human disease genes) and *drug target genes *(consisting of 749 drug target genes in the current knowledge base).

**Figure 5:**
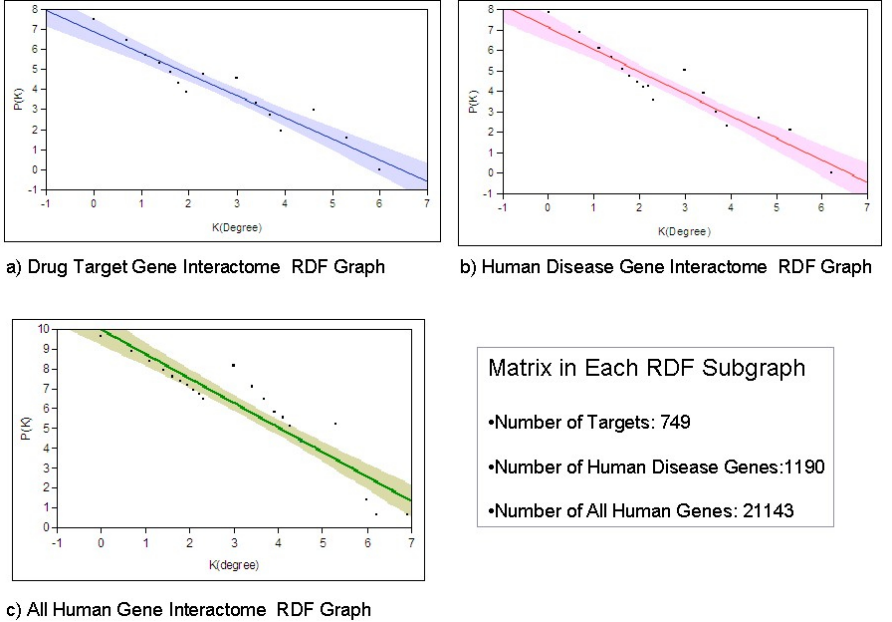
**Scale-free topological properties of gene interactome RDF subnetworks**. Log-Log plot of degree distribution for subgraphs of: a) Drug target genes (linear fit: LogP(K) = 12.89 - 2.64* LogK_(*degree*)_). b) Human disease genes (linear fit: LogP(K) = 13.47 - 2.76*LogK_(*degree*)_). c) All human genes (linear fit: LogP(K) = 9.98 - 1.23* LogK_(*degree*)_).

We observed a general scale-free degree distribution in both drug-centric and gene-interaction subnetworks. In scale-free network, the degree distribution follows power laws, denoted as *P(k) *∞ *K*^-*r*^, with *P(k) *denotes the degree distribution of nodes with degree *k*. Specifically, the degree distributions of the drug-target and drug-indication subnetworks are power law with exponents of 1.32 ± 0.05 and 1.20 ± 0.03, respectively. The interactome subnetworks for disease genes and drug target genes have similar exponent values of 2.64 ± 0.10 and 2.76 ± 0.12, respectively. These values are much greater than the exponent for the overall human interactome network (1.23 ± 0.09) (see Figure 5), suggesting a stronger preferential attachment model for both drug target genes and disease genes compared with average human genes. That is, a stronger "hub" effect may exist in drug target genes and disease genes regards to their interactions with other genes.

### A motivating scenario from Tamoxifen to SLE

Using a real world use case, we investigated whether we can leverage on our semantically integrated comprehensive knowledge base to find complex associations supporting drug repositioning for Systemic Lupus Erythematosus (SLE), a chronic immuno-inflammatory disease that exhibits strong gender bias and the tendency to affect multiple organ systems including heart, skin, joints, kidney and nervous systems. At the current stage, there is no cure for SLE and treatments are largely limited to the relief of symptoms and limited ability to protect organs from inflammation or autoimmune activities in the body by using drugs such as nonsteroidal anti-inflammatory agents or corticosteroids.

#### Ranking drug candidates for SLE using centrality analysis

We issued RDF query to constructed SLE-centric RDF subnetwork which consisted of two level of information: 1) the primary genes associated with SLE as well as all the associated annotations for these primary genes including pathway, gene ontology association, interacting entity, and drug targeting on these genes; 2) the secondary genes interacting with or participating in the sharing pathway and biological process with above primary genes as well as associated annotations (GO, pathway, interacting gene, disease, drug) for these secondary genes. With the semantic representation of multi-source biomedical data in our knowledge base, the entities are encoded according to S-V-O (subject-verb-object) triples with *Subjects *and *Objects *as nodes (e.g., genes, pathways, diseases, symptoms) and lines labeled with *Verbs*. This readily allowed us to perform systemic network analyses using centrality algorithms and graph-based approaches. By applying betweenness and closeness centrality ranking metrics on the resultant subnetwork, we found Tamoxifen was consistently ranked 1^st ^using both ranking approaches (See Additional file 1: Supplement Table), suggesting its candidacy as a modifier and attenuator of SLE disease processes. Tamoxifen, one of the selective estrogen receptor modulators with tissue-specific activities, is an FDA approved-drug for breast cancer treatment and prevention. It acts as both anti-estrogen (i.e. in the mammary tissue) and estrogen-stimulating effects (i.e. in cholesterol metabolism, bone density, and cell proliferation in the endometrium). By manually searching for evidence of this identified association, studies demonstrating the beneficial effects of Tamoxifen on SLE have been observed in animal studies [25], excluded from our data sets. Preliminary clinical studies have also demonstrated that there are subsets of lupus patients with an estrogen-exacerbated disease and one proposed hypothesis is that selective estrogen receptor modulators such as Tamoxifen may have therapeutic potential in SLE patient management [26,27].

#### Identifying biological entities of importance underlying SLE-Tamoxifen association

Undoubtedly, solid validation of specific drug-disease associations predicted using *in silico *approach would await results from extensive animal model testing and human clinical studies. However, by extending the semantic mining and ranking techniques, possible mechanisms with supporting evidences can be inferred to facilitate hypothesis generation and guide further study design. While the pathogenesis of SLE is very complex and remains unclear, we attempted to test whether our integrated RDF semantic knowledge base would be able to uncover the implicit links between Tamoxifen and SLE. First, we issued RDF queries to retrieve Tamoxifen and SLE RDF subgraph respectively:

• For Tamoxifen: *Retrieve all genes and their annotation (interacting gene, pathway, and gene ontology) that are associated with Tamoxifen by acting as its drug target(s) or indication(s)*

• For SLE: *Retrieve disease genes, or genes interacting with or sharing pathways with SLE disease gene as well as their annotation*

**Figure 6:**
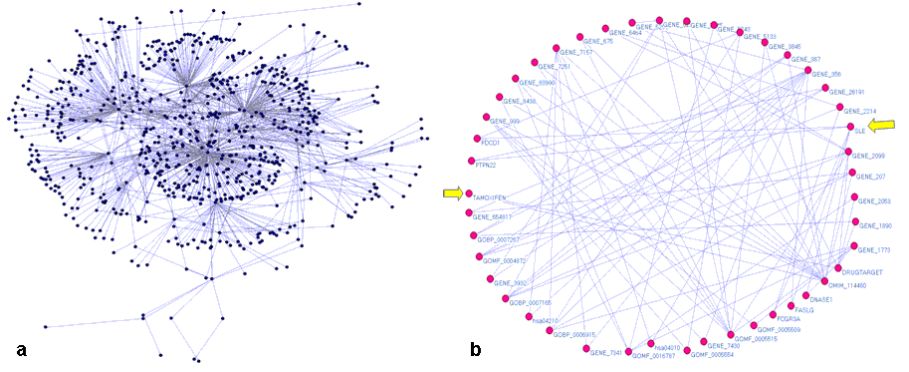
**Graphic view of Tamoxifen-SLE RDF triple network**. (a): Combined RDF graph of all biological entities (e.g. genes and interacting genes, pathways, diseases, and gene ontology terms and inferred annotations) associated with Tamoxifen or SLE. (b): All shortest paths connecting "Tamoxifen" and "SLE". The shortest paths between entities of Tamoxifen and SLE consist of 45 nodes, with minimal distance of 6 measured in geodesic distance. The entities of "Tamoxifen" and "SLE" are highlighted with block arrows.

**Figure 7:**
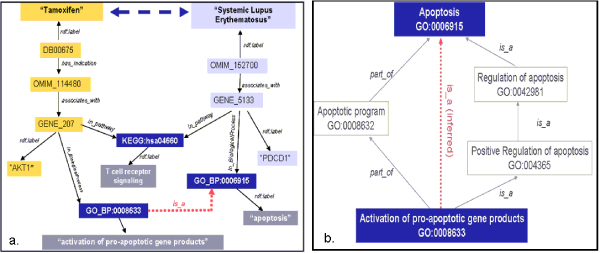
**Example of one shortest path associating Tamoxifen and SLE exploited from SLE-Tamoxifen network**. (a): An association between drug Tamoxifen and disease SLE derived from using DDCO-guided RDF query and inferencing. Arrowed line denotes the connection path between data instances. Red dotted line shows the transitive association inferred from using rulebases in the model. (b): Schematic view showing the graphic relations between GO terms: GO_0006915 (annotation for Gene_5133) and GO_0000008633 (annotation for Gene_207). Using the transitive inference rules created in the rulebases, the association between Gene_5133 and Gene_207 is inferred.

Each query set returned a collection of variable bindings matching to the query parameters and each unique result produced a graph formed from the triples matching the criteria. The components of the resultant RDF subgraph are summarized in Table 1 and the actual graph is shown in Figure 6a. As expected, since the connection between "Tamoxifen" and "SLE" is non-trivial, no association was detected in each individual RDF subgraph. However, by combining the extracted subgraphs and applying inference rules using subsumption relationships as described in Methods section, we were able to extract the implicit connections between the two entities of interest. Figure 6b shows the embedded relations associating Tamoxifen and SLE, which consists of 45 entity nodes with the minimal geodesics of 6 traversing the two entities extracted from the combined SLE-Tamoxifen RDF graph. For example, one of the associations with shortest path between Tamoxifen and SLE is via a common biological process of apoptosis (*GO_0006915*) and T cell receptor signaling pathway (KEGG: hsa04660) that are traversed by two genes: PDCD1 *(Gene_5133, programmed cell death 1) *that is associated with SLE via property of "*associates_with*", and gene AKT1 *(Gene_207, v-akt murine thymoma viral oncogene homolog 1) *that is found to be associated with known indication of Tamoxifen (Figure 7a). Notably, gene PDCD1 is annotated with GO term "apoptosis", yet gene AKT1 is not explicitly annotated with the same GO term. Instead, AKT1 has GO annotation of "*activation of pro-apoptotic gene products*", which is a child and grandchild term of the biological process "*apoptosis*" (Figure 7b). With transitive inference, the association between PDCD1 and AKT1 via biological process was able to be identified and returned to relate Tamoxifen to SLE.

**Table 1:** Number of entities and associations for the resultant RDF subgraphs derived from RDF queries for SLE, Tamoxifen, and Combined

***RDF Graph***	***SLE***	***Tamoxifen***	***Combined***
**Entities**	114	695	768
**Associations**	121	947	1050

As described above, since RDF triples are naturally represented in graph, we sought to use applied graph centrality analysis algorithm and approach [28] to identify the key biological entities within the extracted RDF graphs. As a result, two critical genes were identified with highest ranking impact in both closeness and betweenness measures of the Tamoxifen-SLE RDF graph: ESR1 (*estrogen receptor 1*) and AR (*androgen receptor*). In fact, accumulating evidence from prior studies has suggested a functional crosstalk between immune and endocrine mechanisms in modulating immunity responses [29,30]. Recent studies have reported that estrogen intervention can differentially affect immune cell development via ER (estrogen receptor) signaling under autoimmune or inflammation environment such as inhibits cell survival [31,32]. Rider *et al *has shown that estrogen level alteration in SLE patients appears to affect the development and severity of SLE [33,34]. ESR1, a primary receptor for estrogen, is also a well-understood primary target for Tamoxifen. Tamoxifen binds to ESR1 and causes conformational changes in the receptor which may induce context specific agonist or antagonist activity and thus has the potential to modify estrogen signaling effects on immunity-related disease progression. Contrary to estrogen, androgen deficiency has been shown to be associated with the development of SLE yet the mechanism remains unclear [35-37]. Based on literature mining, both genes identified as well as their dependent genes are found to be differentially expressed in SLE patients and may thus play a role that can alter SLE pathogenesis, disease course, and offer opportunities for improved patient management and therapeutic approaches [26,34,38-40].

## Discussion

Next-generation decision support to optimize the yield of discoveries and benefits from biomedical research, translational medicine, and clinical care will critically depend on our intelligent use of assembled information from multiple sources of facts, knowledge and data. An outstanding opportunity to refine approaches to this next-generation challenge for computational medicine is represented by the search to find drug repositioning opportunities and to develop new understanding of mechanisms that underlie disease. In this study, we have created a comprehensive pharmacome-diseasome infrastructure using Semantic Web-based technology to integrate multiple knowledge sources. By using a specific scenario involved in drug development, we have provided anecdotal evidence for the benefit of such semantic knowledge integration and representation in identifying and ranking drug candidates associated with SLE based on their centrality in the RDF graph. There have been multiple prior efforts to build semantically integrated databases, such as YeastHub [41], FungalWeb [42], and the RDF databases constructed by Sahoo *et al *[43,44] and Stephen *et al *[45]. Our approach differs from these previous efforts in several aspects. First, we have created an integrative RDF data network extending from high dimensional biological-centric data, which most prior work was focused on, to an integrative representation of the pharmacological domain by integrating all FDA-approved drugs and their relationships to the broad spectrum of human disease and their associated clinical features. The integrated data spans biological, genomic, phenotypic, and pharmacologic topologies. Importantly, the resulting RDF is fully anchored by an ontology, the Disease-Drug Correlation Ontology (DDCO), a first draft of a formal and systemic OWL ontology framework we constructed to enable inference and accommodate most current data sources in bioinformatics and chemoinformatics. Second, we used graph-theoretic analyses to examine the topological and relationship properties of drug-disease associations to rank candidate drugs in subgraphs derived from RDF queries. Following this approach, at this stage of our analysis, appears to allow inference of the key entities and processes associated with the resulting semantic network. Even at this early stage, we believe this type of system has the potential to facilitate better understanding of disease mechanism the mechanistic connections for the identified association. To our knowledge, this is the first effort in applying network centrality on the semantically integrated pharmacome-diseasome knowledge base to predict drug-disease associations on the RDF network.

Our hypothesis is that the drugs or genes that are central in the disease-specific RDF subnetwork are likely to be related to the disease. Our results provide supporting evidence for the hypothesis. As shown in the result, ESR1 has been identified as one of the key genes in the SLE-Tamoxifen data network based on the centrality measurements. Estrogen receptor signaling has been shown to regulate differentiation and maturation of immunity cells and impact the immunity modulation by cross talking other pathways [46]. At molecular level, estrogen is found to activate lupus T cells in vitro and estrogen level is elevated during peak time of SLE onset in human studies [33,34]. Our finding has strengthened the hypothesis that estrogen receptor signaling could play a role in autoimmune disease such as SLE. By modulating this pathway using SERM molecules may present novel opportunities for prevention and treatment for SLE. Notably, a very recent clinical study has evaluated Fulvestrant (Faslodex, AstraZeneca Pharmaceutical), also a SERM drug, in therapy of women with SLE and the results demonstrate significant improvement of SLE disease activity and reduction of T cell activation marker [47]. Being a non-patent SERM drug, Tamoxifen confers significant advantage over newer drugs in repositioning to SLE in being inexpensive and well-tolerated with known side effect profile. Another interesting gene with high importance based on centrality analysis in this work is androgen receptor (AR). It has been recognized that androgen deficiency can predispose to and accelerate lupus progression [36], suggesting androgen alone may have therapy utility in SLE. Intriguingly, though AR has not yet been shown to be a direct target for Tamoxifen, positive binding of Tamoxifen to AR has been shown in the receptor binding assay [48]. Furthermore, some studies have suggested that Tamoxifen not only binds to AR but also inhibits AR activity [49,50]. In addition, recent findings using rhesus monkey model have demonstrated Tamoxifen has androgen-like effects on primate mammary sex steroid receptor suggesting the protective action of Tamoxifen may also involve androgenic effect [51]. The interactions between the hormonal signaling pathways and their interacting pathways as well as the effect of pharmacological modulation remain inadequately understood. We have attempted to retrieve the complex and implicit associations between Tamoxifen and SLE using ontology-guided RDF queries, demonstrating the capability of using our semantic infrastructure in inferencing non-trivial relationships. The associations identified, such as apoptosis process (Figure 7), would provide basis for hypothesis generation in suggesting and elucidating connections underlying pharmacological action of therapeutic options in SLE.

The knowledge model and approach in data mining and ranking demonstrated in this study can be generalized to more complex diseases and to additional information sources. The DDCO constructed in this work has provided a formalized ontology for integrating biological and pharmacological knowledge domains. It is designed with flexibility and extendability in mind by providing well-structured upper-level schema scaffold readily for integrating additional knowledge components to accommodate new type of data instances. Further, the equivalence specifications defined in the DDCO can assist with uniting data if different identifiers were initially used. With the continuous enrichment of functional annotations in biomedical areas including disease, gene, pathway, and molecule properties, we envisage a proportional increase of the usefulness and performance of such semantic infrastructure. Nevertheless, how the platform efficiently collects and transforms large amount of new data from heterogeneous biological sources and appropriately mapping ontology content in an efficient manner remains an issue to be solved. While some of the major data sources become available in RDF/XML format, such as UniProt, GO, NCBI Taxonomy, an improved and consistent data conversion/distribution mechanism and system, such as Bio2RDF project under development for making biological data available in RDF document [52], will be beneficial to the scientific community in constructing and expanding semantic knowledge repositories.

Our approach however has some limitations. First, in this work, the centrality measures of closeness and betweenness were chosen to rank the biologically significant entities since they don't require to pre-assign weight values to entities (i.e. to reflect the *usefulness *of nodes) like many other centrality measures. We recognized other centrality measures, such as eigenvector-based centrality, may provide additional power in refining entity ranking. Indeed, we have applied PageRank, a modified eigenvector centrality algorithm underlying the popular search engine Google [53] in disease gene ranking against our semantic knowledge base which has led to very promising findings [54,55]. At the current stage when we only have sparse understanding in the disease-drug mechanisms to define the optimal eigenvalues, we consider the approach of using closeness and betweenness algorithms helps avoid arbitrary bias in ranking. It's no doubt that comparing these different graph theory-based centrality algorithms in ranking biomedical entities, including disease causal genes or drug candidates likely intervening disease process, would be valuable in improving and refining hypothesis generation for biomedical researchers. Second, we have relied on traversal algorithms on in-memory graph representations for subgraph extraction in detecting the key associations underlying entities such as between SLE and Tamoxifen. The performance may be impeded when handling massive graphs. The research advancement in developing extended RDF path query language that supports not only pattern matching but also subgraph extraction by introducing path variable parameters, such as SPARQ2L [56], provides a promising avenue to overcome such limitation.

## Conclusion

We have presented a novel OWL-formalized ontology framework for use in biomedical and pharmacological domain applications. We show that by implementing an integrated pharmacome-diseasome RDF network based on this framework that the DDCO, a goal-driven architecture, is effective in knowledge acquisition, integration, and inconsistency resolution, and data interrogation. The application scenario we presented in this paper illustrates that the DDCO framework and its supported RDF graph data model, in combination with ontology-guided mining and network analysis, could play an important role in an exploratory context in forming or validating hypotheses. Our results strongly suggest that a knowledge framework capable of traversing the spectra of therapeutic agent mechanisms and disease pathophysiological processes can provide a powerful tool for both drug development (see our prior work [54,57]) and support the identification of new disease applications for existing therapeutics.

## Methods

### Ontology development

We used a manual construction and import approach to provide broad coverage across the breadth of disease and drug knowledge. The ontology editor *Protégé *[58] was used as the primary tool for implementing an OWL framework. Previously existing ontologies were thoroughly examined to select relevant reusable knowledge resources to allow efficient knowledge mapping and sharing among independent data sources. Ontology alignment, i.e. mapping between concepts from two or more ontologies or vocabularies, were carried by using PROMPT tool [59]. In addition, manual modifications, such as pruning irrelevant or duplicate branches or adding new concepts/relationships, were performed to accommodate integration needs and minimize incompatibility.

For knowledge domains lacking high quality ontology or mature standards, we sought to build our own ontology by applying approach analogous to CRISP-DM methodology [60] and as described by [61]. One of the key steps of ontology development is to comprehensively and accurately define the relationships between entities. This remains a challenging task since most of the entities in DDCO are extracted from numerous knowledge resources whose relationships are poorly defined. We chose to manually curate the relationships and define domain and range constraints for concepts and properties to support the inference capability of the infrastructure. We used RACER [62], a description logic reasoning system with support for T-Box and A-box reasoning, to pose DL queries for the ontology evaluation. On average, the subsumption computations were completed within seconds and we sought to solve any inconsistencies to assure the integrity of the DDCO.

### Knowledge and data sources

DrugBank is a freely available public resource and the data used for this work was downloaded in flat file format from . Data from UMLS knowledge source was downloaded from . Gene Ontology was downloaded from Gene Ontology website .

Corresponding human gene and GO annotation were downloaded from NCBI Entrez site . Pathway annotation was compiled from KEGG , BioCarta , BioCyc , and Reactome download site . Online Mendelian Inheritance in Man (OMIM) records in XML format and the corresponding gene associations to OMIM disease were extracted from "*mim2gene*" file in NCBI ftp site.

### Mapping associations between pharmacome-diseasome

**Figure 8:**
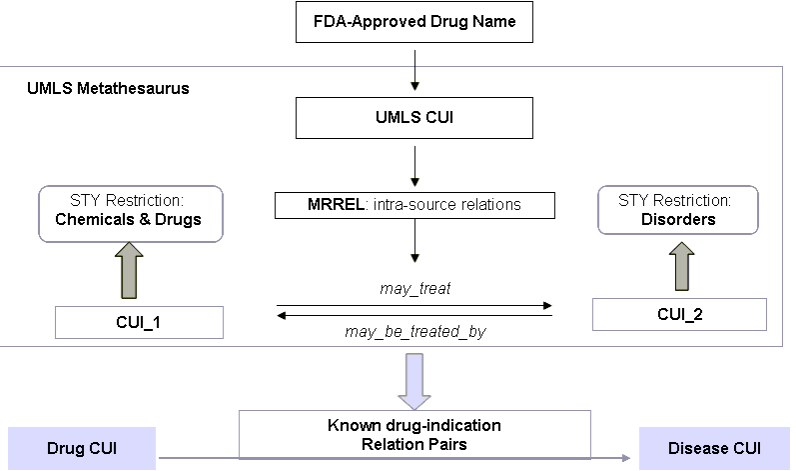
**Schematic diagram workflow for extracting indications for FDA-approved drugs**. MRREL: the primary Metathesaurus file defining intra-source relationships in UMLS; STY: semantic type; CUI: concept unique identifier for concepts in UMLS; "may_treat" and "may_be_treated_by" are UMLS semantic relations used to refine the drug-indication extraction.

To explore the implicit associations between drug and disease, we need to understand the "explicit" relationships between them, i.e. known or approved indications for the drugs of interest. For FDA-approved drugs, the most accurate and comprehensive resource for such information would be the drug labeling system adapted by FDA. However, such label information is commonly embedded in the product document in a free-text or semi-structured manner. Instead of using natural language processing approach to extract such label information, which is error-prone and requires significant manual revision, we chose to extract the drug-indication associations from UMLS Knowledge Server. We used the table MRCONSO.RRF (version UMLS 2007 AC), which is the primary Metathesaurus relationship file defining intra-source relationships in UMLS, to map FDA-approved drugs to the UMLS concepts, each with a unique Concept Unique Identifier (CUI). The table MRREL.RRF, the primary file providing the intra-source relationships of non-synonymous concepts, was used to extract the associated indications for these drug CUI concepts. There are total of 266 distinct relations represented in the MRREL.RRF table, with one row for each relationship between concepts or atoms of UMLS. To extract the drug-indication relationship pairs, we used the relations of *"may_treat" *and *"may_be_treated_by"*, which represent both directions for a relationship (i.e. Concept_1*"may_treat" *Concept_2; Concept_2 *"may_be_treated_by" *Concept_1). To further refine the extraction and eliminate false positive mapping, the semantic type "Chemicals & Drugs" and "Disorders" were used to constrain the returned association concepts (see Figure 8). As a result, a total of 230,114 drug-indication associations were extracted. The set was further refined to 4,413 indications (i.e. "Disorders") for the 1,421 FDA-approved drugs of interest, with both diseases and drugs mapped to the concept unique identifiers (CUI).

### Constructing RDF network

JENA API , a java-based framework for building semantic web applications and supporting RDF(S)/OWL, was used to generate triples required for RDF in compliance with the definitions by the DDCO model. The data includes drug information (drug, target gene/protein, dosing, ingredient, formula, and pharmacological features), genomic data (gene, pathway, gene ontology association) and disease information (disease and associated genes, therapeutic options). The RDF triples from extracting above mentioned pharmacome and diseasome data sources were further converted into N-Triple format using Oracle RDF loader and then loaded to the Oracle 10g release 2 RDF data store [16].

### Data mining and construct subnetwork using RDF query and inference

We used SPARQL and SPARQL-like RDF query syntax such as SDO_RDF_MATCH table function that is required by the Oracle RDF data model to query the data in our RDF data network. The query attributes consists of given RDF graph to be searched, i.e. SDO_RDF_MODELS created in Oracle database, and query which is a SPARQL-like graph pattern containing a set of variables. Each query returns a set of variable bindings matching to the query parameters and the results produces a graph formed from the triples meeting query criteria. To construct RDF subnetwork for each query result, for example, SLE-centric data, the query form of "CONSTRUCT" from SPARQL was used to extract the associated graph for each query result. We generated the inference rules in our rulebase to support RDF inferencing query following below definitions [44]:

• *IF <A is_a B> AND <B is_a C>, THEN <A is_a C>*

• *IF <A is_a B> AND <B part_of C>, THEN <A part_of C>*

• *IF <A part_of B> AND <B is_a C>, THEN <A part_of C>*

• *IF <A part_of B> AND <B part_of C>, THEN <A part_of C>*

Oracle 10g store supports RDFS and custom developed inference rules. We implemented the rulebases for inferencing and created rule indexes enabling pre-computed triples to be inferred from the models built in Oracle 10g.

### Network centrality analysis

Degree centrality was used to examine the general topological properties of the integrated RDF network. In a network, the degree of a node is one of the measurements of the centrality of a node. It's defined as the number of lines connecting to the nodes [24]. The degree K_i _of node *i *is calculated as follow:

$$\text{Ki}=\sum_{j=1}^{n}A_{ij}$$

where *A*_*ij *_= 1 if there is an edge between node *i *and *j *and *A*_*ij *_= 0 if there is not edge connecting *i *and *j*.

The mean degree (K) of nodes in a network is computed by:

$$\text{K}=\frac{1}{N}\sum_{i=1}^{n}Ki(Gn)$$

where *k*_*i*_*(G*_*n*_*) *is the degree of node *i *in graph *G *consisting of *n *nodes.

The degree centrality of a network is associated measure of centralization for the entire network, which expresses the extent to which a network has a center. It is calculated by the variation in the degrees of nodes divided by the maximum degree variation which is possible in a network of the same size [63].

The importance of the entities in the network is evaluated using closeness and betweenness centrality algorithms and analyses. Closeness evaluation emphasizes the distance of one entity to all others in the network, i.e. the smaller the total distance of a node to other nodes, the higher its closeness is. Betweenness centrality calculation balances the importance ranking by weighing in the global importance of an entity that assesses the proportion of the shortest paths between all entity pairs in the network that pass through the entity of interest, i.e., an entity is ranked high of importance if more entity pairs in the network are connected dependent on this entity. The average of the two calculations is used for final ranking. For a graph G = (V, L), where V is the set of *nodes *and L is the set of lines (links, edges), the two parameters is computed as below. [24]

$$\text{Closeness}\left(\text{v}\right)=1/\sum_{w\in V}dist(v,w)$$

- *dist(v, w) *denotes the length of a shortest path between the nodes *v *and *w *in the set of nodes *V *[64]

$$\text{Betweenness}\left(\text{v}\right)=\sum_{\begin{array}{l} s\neq v\neq t\\ s,v,t\in V \end{array}}\sigma st(v)/\sigma st$$

-*σ*_*st *_is the number of shortest paths from node s to t and *σ*_*st*(*v*) _is the number of shortest paths from s to t that pass through the node v.

## List of abbreviations used

DDCO: Disease-Drug Correlation Ontology; SLE: Systemic Lupus Erythematosus; SW: Semantic Web; OWL: Web Ontology Language; RDF: Resource Description Framework; GO: Gene Ontology; UMLS: Unified Medical Language System; W3C: World Wide Web Consortium; XML: eXtensible Markup Language; SERM: Selective Estrogen Receptor Modulator; ADME: absorption, distribution, metabolism, excretion

## Competing interests

The authors declare that they have no competing interests.

## Authors' contributions

AXQ conceived and implemented the use scenario in this research work and is the primary author for drafting the manuscript. AXQ and RCG carried the semantic infrastructure design and implementation. EKN participated in the design and discussion of the research and helped to draft the manuscript. BJA directed and designed the research project and contributed to writing the manuscript. All authors read and approved and final manuscript.

## Supplementary Material

Additional file 1 Supplement Table: Top 10 Ranked Drugs Associated with SLE by Closeness and Betweenness Centrality Measurements. Click here for file
