# Supplementary material for: Inferring novel disease indications for known drugs by semantically linking drug action and disease mechanism relationships
Source: BMC Bioinformatics. 2009 May 6;10(Suppl 5):S4. doi: 10.1186/1471-2105-10-S5-S4 (PMC2679404; doi:10.1186/1471-2105-10-S5-S4)
Supplement: Additional file 1 — Supplement Table: Top 10 Ranked Drugs Associated with SLE by Closeness and Betweenness Centrality Measurements. [file 1471-2105-10-S5-S4-S1.doc]

**Supplement Table: Top 10 Ranked Drugs Associated with SLE by Closeness and Betweenness Centrality Measurements**

| ***Rank*** | ***Closeness*** | ***Betweenness*** |
| --- | --- | --- |
| 1 | **Tamoxifen** | **Tamoxifen** |
| 2 | Inositol 1,3,4,5-Tetrakisphosphate | N,O6-Disulfo-Glucosamine |
| 3 | Cis-[4,5-Bis-(4-Bromophenyl)-2-(2-Ethoxy-4-Methoxyphenyl)-4,5-Dihydroimidazol-1-Yl]-[4-(2-Hydroxyethyl)Piperazin-1-Yl]Methanone | Pentosan Polysulfate |
| 4 | Cis-[4,5-Bis-(4-Chlorophenyl)-2-(2-Isopropoxy-4-Methoxyphenyl)-4,5-Dihyd Roimidazol-1-Yl]-Piperazin-1-Yl-Methanone | Aldesleukin |
| 5 | Methyl Mercury Ion | O2-Sulfo-Glucuronic Acid |
| 6 | Cetuximab | Imatinib |
| 7 | N-{3-Chloro-4-[(3-Fluorobenzyl)Oxy]Phenyl}-6-[5-({[2-(Methylsulfonyl)Ethyl]Amino}Methyl)-2-Furyl]-4-Quinazolinamine | Interferon gamma-1b |
| 8 | Gefitinib | 1na |
| 9 | Erlotinib | Prednisone |
| 10 | Paclitaxel | Interferon beta-1b |
